# Health and greenhouse gas mitigation benefits of ambitious expansion of cycling, walking, and transit in California

**DOI:** 10.1016/j.jth.2017.04.011

**Published:** 2017-09

**Authors:** Neil Maizlish, Nicholas J. Linesch, James Woodcock

**Affiliations:** a Berkeley, CA, USA; b Los Angeles, CA, USA; c Centre for Diet and Activity Research (CEDAR), UKCRC Centre for Diet and Activity Research, Institute of Public Health, Cambridge CB2 0SP, United Kingdom

## Abstract

The purpose of this research was to quantify health co-benefits and carbon reductions of preferred scenarios of California regional transportation plans and alternatives with ambitious levels of active transport. The alternatives were designed to examine the efficacy of independent contributions of walking, bicycling, and transit at levels consistent with the U.S. Surgeon General recommendation for physical activity. Using data from travel and health surveys, vital statistics, collision databases, and outputs from regional and statewide travel models, the Integrated Transport and Health Impacts Model estimated the change in the population disease burden, as measured by deaths and disability adjusted life years (DALYs), due to a shift from a 2010 baseline travel pattern to an alternative. Health pathways modeled were physical activity and road traffic injuries. The preferred scenarios increased statewide active transport from 40.5 to 53.4 min person^−1^ w^−1^, which was associated with an annual decrease of 909 deaths and 16,089 DALYs. Sensitivity analyses that accounted for 2040 projected age- and sex-specific population characteristics and cause-specific mortality rates did not appreciably alter the annual change in deaths and DALYs on a population basis. The ambitious, maximal alternatives increased population mean travel duration to 283 min person^−1^ w^−1^ for walking, bicycling, or transit and were associated a reduction in deaths and DALYs from 2.5 to 12 times greater than the California preferred scenarios. The alternative with the largest health impact was bicycling 283 min person^−1^ w^−1^ which led to 8,543 fewer annual deaths and 194,003 fewer DALYs, despite an increase in bicyclist injuries. With anticipated population growth, no alternative achieved decreases in carbon emissions but bicycling had the greatest potential for slowing their growth. Alternatives that included transit similarly reduced carbon emissions, but with less health benefit. Aggressive expansion of active transport is an efficacious, but underutilized policy option with significant health co-benefits for mitigating greenhouse gases.

## Highlights

• A model estimated health co-benefits and carbon reductions for active transport.
• Health pathways modeled were physical activity and road traffic injuries.
• Scenarios evaluated increased walking, bicycling, or transit to meet health goals.
• Active transportation contributed to significant reductions in disease and injuries.
• Health benefits of official planning scenarios were an order of magnitude lower than optimum.
• Bicycling generated the greatest health benefit per ton of carbon avoided.

## 1 Introduction

Reducing greenhouse gas emissions (GHGE) in the transportation sector is a major thrust of California's response to climate change (California Air Resources Board, 2010, California Air Resources Board, 2014b). Transportation-related GHGEs accounted for 36% of California's 2014 GHGE inventory, and personal passenger vehicles accounted for 70% of the transportation total (California Air Resources Board, 2016). California's multi-pronged policy framework for achieving its carbon mitigation goals in the transportation sector combines fuel efficiency, promoting low carbon fuels, converting California's car fleet to electric vehicles, (Governor's Interagency Working Group on Zero-emission Vehicles, 2013) reducing vehicle miles traveled (VMT) through land use that promotes location-efficient housing, and market mechanisms (Cap & Trade).

The *Sustainable Communities and Climate Protection Act of 2008 (*Steinberg, 2008*)* obligates California's regional transportation planning agencies (called metropolitan planning organizations, MPOs) to meet specific per capita carbon reduction goals through land use strategies (California Air Resources Board, 2010). These include density, land use mix, job-housing balance, network connectivity, regional destination access, and transit access. California MPOs use simulation models to quantify GHGE reductions and other travel outcomes, taking into account decadal economic and demographic forecasts. A range of strategies are modeled, and, after public input, each MPO advances a preferred Sustainable Communities Strategy (SCS) that meets regional goals, but is constrained by foreseeable financing. The preferred SCS undergoes regulatory review by the lead state agency for climate change, the California Air Resources Board, which examines the outputs of the MPO models for the reasonableness of assumptions regarding GHGE reductions from land use. The preferred SCS of each MPO is submitted as part of an environmental impact report (EIR) under the California Environmental Quality Act. Potential harms to human health from air pollution are often included in the EIR and have been a major focus of state agencies and environmental researchers (Zapata et al., 2012). In contrast, quantitative research of the co-benefits or harms of active transportation - walking and cycling - as a greenhouse gas reduction strategy has been less prominent.

Health impact models, such as the Integrated Transportation and Health Impact Model (ITHIM), have enabled an integrated assessment of transportation-health pathways that include air pollution, physical activity, and road traffic injuries (Mueller et al., 2015). Several studies have quantified significant health benefits from increased physical activity in ambitious scenarios of active transportation (Metropolitan Transportation Commission, 2013, Woodcock et al., 2013). These studies suggest far greater health benefits from physical activity than from reduced air pollution, even after accounting for increased injuries of pedestrians and bicyclists and heighted exposure to pollutants by active travelers (Tainio et al., 2016).

There have been few instances of predictive models being used to quantify the health impacts of regional transportation plans in the United States (Whitfield et al., 2016). The purpose of this research was three-fold: 1) to assess the statewide health impact of the preferred SCSs of major California regional transportation planning agencies, 2) quantify regional variability of health and GHGE outcomes, and 3) compare the preferred SCSs with scenarios that illustrate the health and carbon impacts of ambitious levels of walking, cycling, and transit. Previous versions of ITHIM did not model temporal trends in age-, sex-, and cause-specific death rates or population growth and aging. We now contrast health outcomes of models that consider these trends.

## 2 Methods

### 2.1 Model and health outcomes

Previous research has identified physical activity, air pollution, and traffic injuries as the main, direct pathways of transportation-related health co-benefits and harms (Mueller et al., 2015). The Integrated Transport and Health Impact Model (ITHIM) estimates the change in the population disease burden due to a shift from a baseline travel pattern to an alternative with greater active transport. The approach and application to transport and health have been described previously (Metropolitan Transportation Commission, 2013, Woodcock et al., 2013, Whitfield et al., 2016, Fairley and Burch, 2010, Woodcock et al., 2009).

In brief, the model incorporates an extension of the population attributable fraction, which is used in public health to describe the percent of disease or injury that could be avoided in a population by eliminating a risk factor such as lack of physical activity. The population burden of disease was measured in disability adjusted life years, DALYs, which are the sum of years of life lost due to premature mortality and years of living with disability. The population attributable fraction was estimated from exposure-response relationships between a) the risk factor and the health outcome for specific causes, and b) the exposure distribution of the risk factor in the baseline population and in the alternative. ITHIM incorporates specific chronic diseases that have strong evidence from systematic reviews of a relative risk (RR)-exposure gradient for physical activity. These include cardiovascular diseases (ischemic heart disease, hypertensive heart disease, and cerebrovascular disease), colon cancer, breast cancer, diabetes, depression, and dementia, which account for 37% of the burden of disease and injury in the United States (U.S.) (Burden, 2013).

Physical activity encompasses both travel and non-travel related physical activity, including leisure and occupational activities. Daily or weekly activity times were multiplied by weights to give metabolic equivalent task (MET) hours (Ainsworth et al., 2011), which reflect energy expenditures for walking and bicycling at average speeds and for occupational tasks.

For traffic injuries, a distance-based model was used. Injuries were estimated by multiplying change in miles traveled of one or more parties to a collision for each pairwise combination of victim and striking vehicle (pedestrian, bicyclist, motorcycle, car, bus, truck) by the baseline rate per mile traveled for injuries of that combination of modes. A square root function was applied to travel miles to account for the observation that pedestrian and bicyclist injuries tend to be lower at higher mode shares – "safety-in-numbers" (Elvik and Bjørnskau, 2015). Injury risks were stratified by severity (fatal, serious) and roadway type (local, arterial, and highway), which indirectly takes into account the role of speed and traffic volume in traffic injuries.

Because data on air pollution was available for only one California region, this report does not include health impacts due to air pollution. Previous reports for the San Francisco Bay Area and western countries with relatively low ambient concentrations of fine particulate pollution consistently show that active travel generates far greater health benefits from increased physical activity than from lowered air pollution (Niemeier et al., 2015, Metropolitan Transportation Commission, 2013, Teschke et al., 2012). Thus, the lack of statewide modeled data on air pollution for active travel scenarios is not likely to alter the overall findings.

### 2.2 Study population

The study included the 2010 residential population of the five largest California transportation planning regions: San Francisco Bay Area, Sacramento Area, San Joaquin Valley, Southern California, and San Diego County. These regions comprised 30 of California's 58 counties, and make up 97% of the state's population. Rural counties, mostly in the north, east, and central coast, did not have statistically reliable health or travel data, and were excluded from analysis.

### 2.3 Model parameters and data sources

ITHIM has approximately 15 parameters which are means, medians, percents, and a coefficient of variation. They were derived from disaggregated data of travel and health surveys, a collision database, and outputs of regional and state travel demand models.

#### 2.3.1 Baseline travel distances and times

Per capita mean daily distances for walking, bicycling, and motorcycling were calculated from trip lists, and person and household data in the 2012 California Household Travel Survey (CHTS) (NuStats, 2013). Travel distances in the CHTS were based on x-y coordinates of reported origins and destinations and an algorithm in Google Maps for mode-specific travel in a gridded roadway network. Applying national guidelines (Stopher et al., 2008) to CHTS, missing or improbable distances and speeds by mode were imputed for each trip segment using reasonable maximum speed to identify outliers and mean speed from non-missing trip segments. Bicycle and walk trips with zero distance and non-zero travel duration were classified as loop trips, whose distance was estimated by multiplying mean speed on non-missing trip segments by reported travel duration. Minutes of physical activity from walking and bicycling were calculated by dividing known and imputed trip distances by the assumed speeds of 3 and 12 miles per hour, respectively, a convention used in the travel models of regional and state transportation planning agencies. These speeds corresponds to MET weights of 4 and 6, respectively. Travel times reported in CHTS were not used because of likely reporting biases in which respondents inflate times to the nearest 1, 5, or 10 min interval, and include trip preparation and waiting times at signals and intersections involving minimal physical activity.

Mean per capita daily vehicle miles traveled (VMT) for cars and trucks were calculated from trip lists outputs of regional activity-based and 4-step travel demand models (San Francisco Bay Area, Southern California, and San Joaquin Valley) and the California Statewide Travel Demand Model (Sacramento Area and San Diego County) (Statewide Modeling Branch, 2009). For the Sacramento Area and San Diego County, per capita mean daily personal miles traveled for cars and buses were based on CHTS. For motorized modes, the percentage of VMT by roadway type was obtained from the fully loaded networks of the travel models. It was assumed that 75% of walking occurs on local roads and 25% on arterials, and that 53% of bicycling occurs on local roads and 47% on arterials (Dill, 2009).

#### 2.3.2 Health outcomes and non-travel physical activity

Deaths and DALYs were obtained from the 2010 U.S. Global Burden of Disease, GBD (Institute for Health Metrics and Evaluation IHME, 2015). To account for local geographic variation, age-, sex-, and cause-specific deaths and DALYs for each California region were adjusted by the ratio of their counties' and U.S. mortality rates. California death files (2009–2011) and the 2010 California decennial Census population were used to construct 3-year annual average county mortality rates. Weekly duration of non-travel physical activity from leisure and occupational activities were derived from the California Health Interview Survey (UCLA Center for Health PolicyResearch, 2005). Fatal and severe injuries were obtained from a statewide database of traffic collisions on public roads (Safety Transportation Research and Education Center, 2011), which were spatially joined to the California road network to assign the federal facility type classification for each collision. Facility types were re-categorized into local, arterial, and highway.

#### 2.3.3 Temporal trends in mortality rates and population characteristics

We estimated annual death rates in 2040 by applying the age-, sex-, and cause-specific annual percent change projected for U.S. death rates for 2009–2040. These data were reported by Canudas et al. Canudas-Romo et al., (2016), who supplemented traditional life table methods with clinical expert opinion. Cause categories were available for cancer, cardiovascular disease, neurodegenerative diseases, diabetes and injuries. These were applied to the respective ITHIM cause categories: 1) breast and colon cancer, 2) ischemic heart disease, hypertensive heart disease, and stroke, 3) dementia, 4) diabetes, and 5) road traffic injuries. No temporal trends were available for depression, which was assumed to maintain nearly zero death rates. The 2010 U.S. GBD for deaths, years of life lost, and years living with disability were adjusted upward or downward at 2040 by applying the annual percent change in death rates over 30 years.

Age-sex population counts were projected through 2040 for California counties by the California Department of Finance; (Demographic Research Unit, 2014) and county populations were aggregated into MPO regions.

#### 2.3.4 Carbon emissions

Aggregate carbon emissions from cars and light trucks were estimated for each California region from modeled emission factors (g CO_2_eq mi^−1^) in 2010 (California Air Resources Board, 2014). The EMFAC model takes into account the characteristics of the vehicle fleet, fuel type (gasoline, diesel, and electric), and operating conditions. The emission factor in 2010 was multiplied by per capita car VMT and the projected 2040 California population. This yields the carbon emissions at a scenario time horizon from substituting active travel and transit miles independently of automotive technology that lowers the carbon intensity of transportation fuels and propulsion systems.

### 2.4 Scenarios

#### 2.4.1 Preferred SCSs

We obtained the EIR of each major MPO (Gold et al., 2002, Metropolitan Transportation Commission and Association of Bay Area Governments, 2013, Safety Transportation Research and Education Center, 2011, Southern California Association of Governments, 2016, Statewide Modeling Branch, 2009), which projected travel 30 years into the future for car, transit, and active transport from a 2010 centered baseline. Each MPO reported population totals, daily vehicle miles traveled for motorized modes, and the annual or daily number of trips for transit, walking, and cycling for their baseline and at their 30-year future time horizon. To align the preferred scenario for each mode in ITHIM, we multiplied the mode's baseline by the percentagewise change in per capita mean daily distances from baseline to scenario as indicated in the EIRs. Relative changes in travel for heavy goods vehicles were not specifically reported, and we used the 2010 baseline levels as an approximation.

#### 2.4.2 Ambitious scenarios

We constructed four alternatives to a 2010 baseline that reflect ambitious expansions of active transport and/or transit that substitute for car travel, but maintain total miles traveled across all modes.

The "Walk" scenario increased transportation-related walking duration to a population mean of 283 min person^−1^ w^−1^ (median 150 min person^−1^ w^−1^) while holding bicycling, non-travel physical activity, and transit times and distances at their baseline levels. One hundred fifty minutes of weekly physical activity represents the U.S. Surgeon General's physical activity recommendation for adults and corresponds to a level beyond which there are smaller health benefits per additional minute of physical activity (Physical Activity Guidelines Advisory Committee, 2008). An equivalent amount of walked miles was subtracted from the car driver and passengers traveling on local roads and arterials, maintaining the baseline ratio of car occupancy and the baseline ratio of local to arterial car VMT.

The "Cycle" scenario increased transportation-related bicycling duration to a population mean of 283 min person^−1^ w^−1^ while holding walking, non-travel physical activity, and transit times and distances at their baseline levels. An equivalent number of bicycled miles was subtracted from the car driver and passengers traveling on local roads and arterials, maintaining the baseline ratio of car occupancy and the baseline ratio of local to arterial car VMT.

The "Transit" scenario took into account walking and bicycling associated transit trips and an ambitious expansion of transit. Ratios of minutes walked per transit minute and minutes bicycled per transit minute were derived from the CHTS. These ratios were multiplied by an increased transit duration to estimate transit-associated active transport. The total travel time for active transport and transit was constrained to the sum of 283 min person^−1^ w^−1^. The baseline median of transit duration was approximately 18.9 min person^−1^ w^−1^_,_ depending on the region. An equivalent amount of transit, walking, and bicycled miles was subtracted from the car driver and passengers traveling on local roads and arterials, maintaining the baseline ratio of car occupancy and the baseline ratio of local to arterial car VMT.

The "Blend" scenario increased both active travel and transit so that the per capita mean daily duration of walking, bicycling, and transit were equal (~70 min person^−1^ w^−1^). An equivalent amount of transit, walking, and bicycled miles was subtracted from the car driver and passengers traveling on local roads and arterials, maintaining the baseline ratio of car occupancy and the baseline ratio of local to arterial car VMT.

### 2.5 Calculation

Statistical software (Statistical Analysis System, SAS 9.3, and R 3.2.1) was used to calculate ITHIM parameters. (SAS and R programs and the ITHIM Excel worksheets are available on request.) The version of ITHIM used in this study is a spreadsheet implemented in Microsoft Excel. Each region was calibrated for 2010 baseline travel in a separate, standardized, and interactive Excel workbook. Each region's results were pooled for the statewide total. As in previous work, travel-related physical activity was modeled as a log normal distribution and population attributable fractions were estimated in population quintiles for age-sex and cause-specific strata. We present both per capita means and medians to describe central tendency of physical activity on a population basis.

#### 2.5.1 Sensitivity analyses of temporal trends in death rates and population characteristics

Sensitivity analyses were performed on the preferred SCSs compared to their 2010 baseline. Age,-sex-, and cause-specific population attributable fractions were multiplied by a burden of disease that incorporated a) the 2010 or projected 2040 death rates, and/or b) the 2010 or projected 2040 population. The combinations of death rates and populations for sensitivity analyses, *S*, were:

*S*_*0*_: 2010 California population experiencing 2010 death rates
*S*_*1*_: 2010 California population experiencing 2040 projected death rates
*S*_*2*_: 2040 California population experiencing 2010 deaths rates
*S*_*3*_: 2040 California population experiencing 2040 projected death rates.

In the base case, *S*_*0,*_ the burden of disease was calculated using 2010 mortality rates and the 2010 population age distribution, and is the reference for comparisons with the other models. The first sensitivity analysis, *S*_*1*_, assessed the change in health impacts from temporal trends in death rates, holding other inputs constant. *S*_*2*_ assessed the health impacts of temporal changes in population composition, holding other inputs constant. *S*_*3*_ assessed the simultaneous impacts of temporal trends in death rates and population characteristics.

## 3 Results

### 3.1 Travel patterns by mode and scenario

#### 3.1.1 Baseline

At baseline, the California population mean of active transport duration was 40.5 min person^−1^ w^−1^. Active transport and transit comprised 4.9% of distance mode share at baseline (Table 1). There was considerable regional variability in baseline travel patterns. The Bay Area region had the highest per capita weekly mean duration of active transport, which was twice that of the San Joaquin Valley and more than two-thirds higher than the other regions. The Bay Area and Sacramento Area had similarly high levels of per capita weekly walking duration compared to the other regions. The San Francisco Bay Area also had the lowest annual per capita travel distance summed for all modes combined (9,093 mi person^−1^ y^−1^) and Southern California had the highest (13,178 mi person^-1^ y^-1^).

**Table 1:** Per capita personal travel distance and active travel times by mode and scenario, California.

	Annual Distance (mi person^−1^ y^−1^)									Travel Time		
			Car		Transit					Mean (median) min person^−1^ w^−1^		
Scenario	Walk	Cycle	Driver	Pas-senger	Bus	Rail	Motor-cycle	Truck	Total	Walk	Cycle	Total
Baseline												
California, 2010	96	38	6,880	3,503	297	147	34	662	11,657	36.9 (17)	3.6 (2)	40.5 (19)
SF Bay Area^a^	151	61	5,683	1,824	294	354	48	677	9,093	57.8 (30)	5.9 (3)	63.7 (33)
San Joaquin Valley	73	18	6,207	3,257	398	8	23	1,108	11,092	27.8 (10)	1.8 (1)	29.6 (11)
Southern California	85	29	7,967	4,182	304	120	27	464	13,178	32.6 (15)	2.8 (1)	35.4 (16)
San Diego County	77	42	4,841	3,062	235	95	49	1,272	9,673	29.5 (15)	4.0 (2)	33.5 (17)
Sacramento Area	84	61	5,653	4,227	160	33	47	622	10,886	32.4 (15)	5.8 (2)	38.2 (17)
SCS, California	125	57	6,319	3,228	386	244	34	662	11,055	48.0 (23)	5.4 (3)	53.4 (26)
Ambitious Scenarios												
Walk	727	38	6,461	3,291	297	147	34	662	11,657	279 (152)	4 (2)	283 (154)
Cycle	96	2,563	5,205	2,653	297	147	34	662	11,657	43 (21)	240 (133)	283 (154)
Transit	188	72	5,195	2,692	1,923	891	34	662	11,657	73 (35)	6 (3)	79 (39)
Blend	245	981	5,284	2,722	1,178	551	34	662	11,657	105 (48)	84 (48)	188 (96)

a San Francisco Bay Area counties: Alameda, Contra Costa, Marin, Napa, San Francisco, San Mateo, Santa Clara, Solano, Sonoma Southern California counties: Imperial, Los Angeles, Orange, Riverside, San Bernardino, Ventura San Joaquin Valley counties: Fresno, Kern, Kings, Madera, Merced, Stanislaus, San Joaquin, Tulare Sacramento Area counties: El Dorado, Placer, Sacramento, Sutter, Yolo, Yuba

#### 3.1.2 Scenarios

In the statewide SCS pooled from the regions, walking increased 30% and cycling increased 50% (Table 2), which increased the per capita weekly mean to 53.4 min (Table 1). Active transport and transit increased to 7.3% of distance mode share. Per capita mean weekly walking increased by 11.1 minutes, and per capita mean weekly cycling increased by 1.8 min. Per capita mean travel distances increased 42% for transit, and fell 8% for cars. The relative changes in travel varied by mode and region. The San Diego County SCS had the largest increases in walking and cycling (88%). Large increases in transit were planned for Sacramento area. Southern California had large relative increases in bicycle and rail travel. Unlike other modes, car travel was projected to decrease from 7% to 11% across regions.

**Table 2:** Changes in daily travel in California regional transportation planning from a 2010 baseline to 2040.

Mode	Metric	Bay Area	Sacramento Area	San Joaquin Valley	Southern California	San Diego Co.
Walk	Trips/p	+11%	+16%	+31.7%	+27%	+88%
Bicycle	Trips/p	+19%	+11%	+31.7%	+69%	+88%
Car	VMT/p	−9%	−10%	−11%	−7%	−11%
Bus	Trips/p	+40%	+145%	+50%	+7%	+73%
Rail	Trips/p	+40%	+145%	+50%	+94%	+73%

In the Walk scenario, distance mode share for walking increased from 0.8% to 6.2% (Table 1). In the Cycle scenario, distance mode share for bicycling increased from 0.3% to 21.9%. In the Transit scenario, the transit mode share increased from 3.8% to 24.1%. In the Cycle, Transit and Blend scenarios the sum of walking, bicycling, and transit were each approximately 26% of distance mode share. Car distance mode share in the Walk scenario declined to 83.7% from the baseline level of 89.1%. Car distance mode share for Cycle, Transit and Blend was each approximately 68%.

### 3.2 Health benefits and harms by scenario

#### 3.2.1 SCSs

In the model using the 2010 population and 2010 death rates (*S*_*0*_*)*, the statewide SCSs decreased the annual number of deaths by 909 and DALYs by 16,089 (Table 3). The population attributable fraction of active transport was 1.2% or smaller for each major diagnostic group (Table 3). Chronic disease rather than road traffic injures accounted for the largest share of the reduced burden.

**Table 3:** Health outcomes in MPOs' combined preferred scenario with and without projected death rates and population trends, California.

Disease Category	Death Rates and Population									
	2010 (*S*_*0*_)				2040 *(S*_*3*_*)*				Ratio 2040/2010	
	Deaths		DALYS		Deaths		DALYS		Deaths	DALYs
	PAF, %	N	PAF, %	N	PAF,%	N	PAF,%	N		

Cardiovascular Disease	−1.1	−623	−1.1	−9,682	−1.2	−1,107	−1.2	−12,523	1.73	1.25
Diabetes	−1.0	−68	−1.1	−2,343	−1.1	−118	−1.1	−2,874	1.66	1.18
Dementia	−1.1	−174	−1.0	−1,908	−1.1	−397	−1.0	−4,244	2.28	2.21
Depression	0.0	0	−0.2	−814	0.0	0	−0.3	−1,158	–	1.42
Colon Cancer	−0.4	−15	−0.3	−203	−0.5	−29	−0.3	−380	1.85	1.86
Breast Cancer	−0.3	−12	−0.2	−223	−0.4	−19	−0.2	−331	1.61	1.48
Chronic Disease (above)		−892		−15,173		−1,633		−20,949	1.83	1.38
Road Traffic Injuries	−0.5	−17	−0.5	−916	−0.6	−20	−0.6	−948	1.20	1.04
Total		−909		−16,089		−1,653		−21,898	1.82	1.36
Rate × 10^5^ population		−2.5		−44.4		−3.3		−43.4		

PAF, Population Attributable Fraction

DALYs, Disability Adjusted Life Years.

In the model using the 2040 projected population and 2040 death rates (S_3_), the statewide SCSs decreased the annual number of deaths by 1,653 and DALYs by 21,898. In comparing models (S_3/_S_0_), the change in the burden of deaths and DALYs was greatest for dementia and smallest for road traffic injuries. Expressed on a population basis, the change in deaths (and DALYs) was similar whether or not projected population or mortality trends were considered (2.5 vs 3.3 deaths 10^−5^ and 44.4 vs. 43.4 DALYs per 10^−5^).

In the model using the 2010 population and 2040 projected deaths rates (S_1_), the SCSs decreased the annual number of deaths by 689 and DALYs by 11,905. In contrasting models that held population characteristics constant but varied disease rates (S_1/_S_0_), the SCSs in the model with lower disease rates reduced deaths (689/909) and DALYs (11,905/16,089) to a lesser degree. In contrasting models that held 2010 disease rates constant but varied population characteristics (S_2/_S_0_), the SCSs in the model with 2040 population decreased deaths by 2,134 and DALYs by 29,002. The change in the burden of disease for the SCSs was greatest for models with this combination of disease rates and population characteristics.

The health impacts of the regional SCSs appeared to follow their relative increases in active transport and transit (Table 4). Compared to other regions, the SCSs of San Diego and Southern California had the largest increases in active travel and the largest net decrease in DALY rates (exceeding −3 DALYs per 10^−5^). However, these two regions had net positive DALY rates for road traffic injuries. Other regions with moderate to small increases in active transport had smaller decreases in DALY rates. The Sacramento area planned a large transit expansion, but modest increase in active transport; its SCS had large decreases in DALY rates from road traffic injuries.

**Table 4:** Change in number and rate (× 10^5^ population) of deaths and disability adjusted life years from chronic disease and road traffic injuries by California region, preferred SCS scenarios compared to the 2010 baseline.

Change in Burden of Disease	Bay Area		San Joaquin Valley		Southern California		San Diego		Sacramento Area		Total	
	N	*Rate*	N	*Rate*	N	*Rate*	N	*Rate*	N	*Rate*	N	*Rate*
Deaths, Total	−101	*−1.4*	−122	*−2.9*	−581	*−3.02*	−114	*−3.7*	−22	*−0.9*	−940	*−2.59*
Chronic Disease	−88	*−1.2*	−116	*−2.7*	−583	*−3.04*	−124	*−4.0*	−11	*−0.5*	−923	*−2.55*
Road Traffic Injuries	−13	*−0.2*	−6	*−0.1*	2	*0.01*	10	*0.3*	−10	*−0.4*	−17	*−0.05*
DALYs, Total	−2,727	*−37.1*	−2,509	*−59.4*	−8,607	*−44.8*	−2,138	*−69.1*	−781	*−32.9*	−16,763	*−46.2*
Chronic Disease	−2,057	*−28.0*	−2,148	*−50.8*	−8,769	*−45.7*	−2,604	*−84.1*	−270	*−11.4*	−15,847	*−43.7*
Road Traffic Injuries	−670	*−9.1*	−362	*−8.6*	161	*0.8*	466	*15.1*	−511	*−21.5*	−916	*−2.5*

Using 2010 population and death rates, the statewide SCSs had increased number of pedestrian and bicyclist fatalities and serious injuries compared to the 2010 baseline (Table 6). On the basis of injuries per 10^7^ miles traveled, injury rates of pedestrians and bicyclists declined 15% and 19%, respectively, in the SCSs.

#### 3.2.2 Ambitious scenarios

The Walk scenario projected a large decrease in chronic disease deaths, largely due to a 10.3% reduction in cardiovascular disease and an 8.5% reduction in dementia (Table 5). The burden from road traffic injuries increased, primarily due to an absolute increase in the number of pedestrian injuries (Table 6). In other modes, injury numbers declined, and, for each mode, including walking, injury rates per mile traveled declined.

**Table 5:** Change in the burden of disease and injury by scenarios of walking, cycling and transit, California.

Disease Category	Scenario							
	Walk				Cycle			
	Deaths		DALY^b^		Deaths		DALY	
	PAF^a^, %	N	PAF, %	N	PAF, %	N	PAF, %	N
Cardiovascular disease	−10.3	−6,152	−11.5	−103,200	−10.5	−6,223	−13.8	−124,122
Diabetes	−10.4	−742	−11.7	−26,551	−11.2	−794	−14.1	−31,978
Dementia	−8.5	−1,457	−8.1	−16,882	−6.1	−1,048	−6.4	−13,271
Depression	0.0	0	−3.5	−12,355	0.0	0	−4.2	−14,764
Colon Cancer	−4.0	−162	−3.5	−2,679	−4.4	−179	−4.7	−3,555
Breast Cancer	−3.5	−143	−3.1	−3,219	−2.1	−86	−2.0	−2,144
Sum of Above		−8,656		−164,886		−8,331		−189,834
Road Traffic Injuries	17.0	551	15.8	27,800	−6.5	−213	−2.4	−4,199
Net		−8,104		−137,086		−8,543		−194,033
	Transit				Blend			
Disease Category	Deaths		DALY		Deaths		DALY	
	PAF, %	N	PAF, %	N	PAF, %	N	PAF, %	N

Cardiovascular disease	−2.4	−1,401	−2.5	−22,861	−7.7	−4,554	−9.5	−85,938
Diabetes	−2.3	−165	−2.6	−5,838	−8.0	−569	−9.7	−22,077
Dementia	−2.4	−415	−2.2	−4,670	−5.4	−918	−5.3	−10,958
Depression	0.0	0	−0.7	−2,381	0.0	0	−2.5	−9,046
Colon Cancer	−1.0	−41	−0.8	−603	−3.0	−121	−2.9	−2,173
Breast Cancer	−0.8	−31	−0.6	−633	−1.8	−76	−1.6	−1,712
Sum of Above		−2,054		−36,987		−6,238		−131,902
Road Traffic Injuries	−6.2	−203	−7.0	−12,336	−3.8	−125	−2.1	−3,714
Net		−2,257		−49,322		−6,363		−135,616

*Note:* Rounding errors reflected in totals.

a PAF, Population Attributable Fraction

b DALY, Disability Adjusted Life Year

**Table 6:** Annual number and rate^a^ of fatal and serious injuries by mode and scenario.

Victim Mode	Baseline		Scenario									
			SCS		Walk		Cycle		Transit		Blend	
	N	Rate	N	Rate	N	Rate	N	Rate	N	Rate	N	Rate
Walk	2,408	6.91	2,678	5.90	5,633	2.14	1,713	4.91	3,136	4.59	2,903	3.27
Bicycle	905	6.65	1,101	5.36	762	5.60	6,749	0.73	1,186	4.55	3,968	1.12
Bus	33	0.03	42	0.03	33	0.03	33	0.03	155	0.02	102	0.02
Car	7,791	0.21	7,295	0.21	6,164	0.17	4,745	0.17	6,209	0.22	4,909	0.17

a Rate = Number of injuries per 10^7^ mi y^−1^ traveled by injured party (victim) of a road traffic collision.

Of all ambitious scenarios, Cycle had the largest reduction in deaths and DALYs. Cardiovascular disease, road traffic injuries and diabetes were the principal contributors to the decline. There was a large reduction in the number of road traffic injuries for car occupants (Table 6), accounting for the net reduction in DALYs due to traffic injuries. There was a 7.45 fold increase in the number of bicyclist injuries. In comparison to the Walk scenario, the Transit scenario generated 28% fewer reductions in deaths and 36% fewer net reductions in DALYs. Although the reduction in road traffic injuries deaths in the Walk and Transit scenarios was similar, DALYs decreased to a greater extent in the Transit scenario. From baseline, the Blend scenario reduced deaths (by approximately 8%) and DALYs (by approximately 10%) due to cardiovascular disease and diabetes.

Injury risks per mile traveled by mode fell substantially in many scenarios through a combination of reduced motor traffic volume, and, in the case of pedestrians and cyclists, safety-in-numbers benefits. Most notably the number of bicyclist injuries per mile cycled fell by nearly 90% in the Cycle scenario, and pedestrian injuries per mile walked fell by nearly 69% in the Walk scenario.

### 3.3 Carbon emissions by scenario

Carbon emissions from cars were influenced by emission factors and car VMT, which was reduced to different degrees in the SCSs and ambitious active transport scenarios. Regions varied in their emissions factors; the San Francisco Bay Area and Sacramento regions had the lowest (Table 7). Regions also varied in their baseline per capita car VMT with Southern California exceeding the Bay area by nearly 4,000 mi person^−1^ y^−1^ (Table 1).

**Table 7:** Annual automobile carbon emissions and 2010 emissions factors by california region.

Region	2010 Car Emissions Factors,= g CO_2_ mi^−1^	Carbon Emissions (MMTy^−1^)^a^						
		Baseline 2010	Scenario^b^					
			SCS	Walk	Cycle	Transit		Blend
California	–	105.1	115.4	130.9	105.6	105.6		107.3
San Francisco Bay Area	408	16.4	19.4	20.5	16.1	12.5		14.1
San Joaquin Valley	419	11.0	12.7	9.2	9.9	9.7		12.7
Southern California	429	65.8	70.3	81.8	67.9	70.2		70.4
San Diego County	428	6.4	6.9	8.2	6.2	6.4		6.5
Sacramento Area	410	5.5	6.5	7.8	6.2	6.7		6.6

a MMT, Million Metric Tons.

b Scenarios include population growth at 2040, car VMT replaced by increased active travel and transit, and emission factors held at 2010 levels; does not include CO_2_ reductions from reduced congestion and other traffic management practices.

**Fig. 1:**
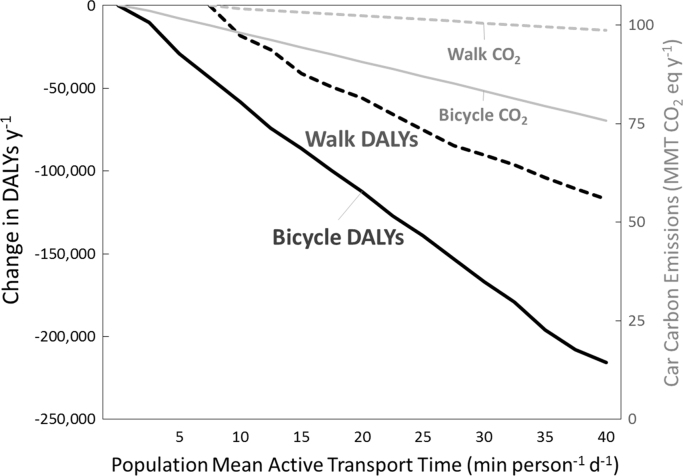
Change in DALYs and CO_2_ reductions per minute of cycling and walking, California 2010 population. Walk DALYs summarize the results of a sensitivity analysis that varied the mean level of walking in the Walk scenario from the state baseline (5.3 min person^−1^ d^−1^) to a high point of achieving the U.S. Surgeon General's recommendations (holding cycling and transit times at their baseline levels.). Bicycle DALYs is the corresponding curve for the Bicycle scenario in which cycling time varied from baseline levels (0.51 min person^−1^ d^−1^) to U.S. Surgeon General's recommendations. The U.S. Surgeon General's physical activity recommendation, expressed as a population mean, is 39 min person^−1^ d^−1^ (median is 22.1 min person^−1^ d^−1^). State transportation agency 2020 goals are approximately 10.6 min person^−1^ d^−1^ for walking and 1.5 min person^−1^ d^−1^ for bicycling. Carbon reductions assume that car miles traveled decrease by an equivalent increase in active travel.

Accounting for statewide population growth at 2040 and holding 2010 CO_2_ emission factors constant, no scenario reduced carbon emissions below the 2010 baseline. The carbon emissions in the pooled SCSs were greater than the Cycle, Transit, and Blend scenarios. The Cycle scenario slowed growth of GHGE from the 2010 baseline and yielded an absolute reduction of 3 million metric tons (MMT y^−1^) from 2000 reference level of 113 MMT y^−1^. The San Francisco Bay Area achieved reductions of 1.6 to 3.9 MMT y^−1^ from its 2010 baseline in the Cycle, Transit, and Blend scenarios. San Diego County carbon emissions fell below the 2010 baseline only in the Cycle scenario (−0.2 MMT y^−1^). The health and carbon mitigation efficacy of the Walk and Cycle scenarios per mean minute of active transport for California are shown in Fig. 1 along with state and regional targets for active transport and bicycling (Elvik and Bjørnskau, 2015, Mueller et al., 2015).

## 4 Discussion

### 4.1 Significance of findings

In the United States, physical inactivity is estimated to account for 6% of all deaths and approximately 30% each of ischemic heart disease, diabetes, colon cancer, and breast cancer, 13% of cerebrovascular disease (Institute for Health Metrics and Evaluation, 2014) and 21% of Alzheimer's disease (Barnes and Yaffe, 2011). For the corresponding causes assessed by ITHIM, physical activity may account for approximately 23,000 California deaths in 2010. We found California's proposed regional transportation plans to reduce the annual number of these cause-specific deaths by 892. This is a positive, but small contribution to improving population health.

Health benefits of the regional SCSs tended to follow the proposed relative increases in active transport and transit. The increase in walking and cycling of the regional preferred scenarios do not appear to be on a trajectory to meet strategic management goals of the California Department of Transportation, which aims to double walking and triple cycling from a 2010 baseline by 2020 (California Department of Transportation, 2015). Likewise, the Bay Area MPO established a goal of 15 min. person^−1^ d^−1^ of active transport; but, the preferred scenario achieved only two-thirds.

Incorporating mortality and population projections increased the absolute magnitude of health impacts, but marginally changed the impacts expressed per population. The change in deaths appeared to be more sensitive to temporal trends than the change in DALYs. This appears to be result of the large share of deaths at ages 70 and older and exponentially increased death rates in this age group. DALYs are less concentrated in ages 70 and older and increase more slowly from younger ages. As the California population ages, a larger proportion of deaths and DALYs will occur in the oldest age groups. By 2040, California will double the proportion of the population aged 70 years or older compared to 2010 (16.3% vs 7.8%).

The ambitious scenarios increased per capita mean weekly active travel to 283 minutes, which is similar to the time burden of 272 minutes experienced by California commuters in 2010 (American Community Survey, 2012). Each ambitious active transport and transit scenario significantly reduced the burden of disease and injury in California. The Cycle and Walk scenarios, in which half the California population meets the U.S. Surgeon General's physical activity recommendations, would reduce the burden of cardiovascular disease and diabetes by more than 10%.

That the Cycle scenario surpassed the others in health benefits is a consequence of the greater intensity of physical activity of bicycling compared to walking (6 vs. 4 METs) for a fixed, mean duration of 283 min person^−1^ w^−1^. Under this scenario there was a substantial increase in bicyclist injuries, but to a much smaller extent than the increase in distance cycled. The Cycle scenario also generated the greatest benefit in carbon reduction. While not leading to an absolute reduction from the 2010 baseline in each region, the Cycle scenario slowed the statewide growth in carbon emissions to the greatest extent. The Transit scenario generated the least health benefit of the ambitious scenarios. The Walk scenario generated substantial health benefits despite increasing pedestrian fatalities. The Walk scenario was the least efficacious in reducing carbon levels because walking covers far shorter distances than bicycles or transit in scenarios constrained by time and a 1:1 replacement of car distances by walking.

The San Francisco Bay Area was the only region that demonstrated consistent reductions in carbon emissions in the Cycle, Transit, and Blend scenarios, and its characteristics may be instructive for identifying the critical pathway to lowering carbon emissions. The San Francisco Bay Area had a less polluting car fleet, but also had the highest per capita baseline of transit and active transport, and a low per capita car VMT. The Sacramento Area and San Diego County had lower per capita car VMT than the San Francisco Bay Area, but lower per capita transit mileage. Despite meeting per capita targets (California Air Resources Board, 2010), the SCSs do not achieve absolute carbon reductions. Much higher levels of active transport and transit are needed to reach this goal.

### 4.2 Strengths, limitations, and assumptions

The strengths, limitations, and assumptions in ITHIM have been discussed in previous publications (Metropolitan Transportation Commission, 2013, Woodcock et al., 2013, Woodcock et al., 2009). In brief, ITHIM incorporates definitive health outcomes on disease-specific deaths and DALYs for which the physical activity-response gradient are well established. We also included DALYs as a key health outcome. DALYs add information on the impact of life shortening and morbidity on the burden of disease. We were able to leverage significant public data and analytic resources from the Global Burden of Disease (GBD) Project (Institute for Health Metrics and Evaluation, 2014). We acknowledge that researchers have raised methodological concerns with DALYs as a metric for health impact modelling regarding reference populations, disability weights, and other issues, and have proposed alternatives (Gold et al., 2002). In the GBD Project years of life lost are combined in DALYs, and it is implicitly assumed that years of life gained are in full health. Age-adjusted years of life lost would help overcome this potential for overestimation of health benefits. However, this metric is no longer produced by the GBD study due to concerns about an inappropriate lower valuation of life gained at older ages. Despite its limitations the GBD has the major advantages of comparable estimates being developed for the whole world, with more detailed estimates being provided with each update.

The injury module incorporates "safety-in-numbers" and stratifies at-risk miles by roadway type because risks and exposure differ considerably by roadway type. There is good evidence to support non-linear risks with variation in distance for many modes (Elvik and Bjørnskau, 2015). However, the most statistically robust evidence is cross-sectional, and, as such, assessing causality is problematic.

There were several limitations in the available data. As reported in EIRs, motorized VMT was not disaggregated by mode, but relative increases were reported for bus and rail travel. We derived per capita car VMT from the total motorized VMT by applying the relative increases for bus and rail to their baseline levels and assumed per capita truck VMT did not change. If per capita truck VMT were higher, the per capita car VMT would be lower. This would have led to a more beneficial effect on road traffics injuries than we reported. For transit, walking, and cycling, travel was expressed as number of trips rather than miles traveled. To estimate travel distances for these modes, we assumed that average trip length did not change over time. For the San Joaquin Valley, motorized travel and the SCS preferred scenario were based on accessible data of a single county's travel demand model output that was scaled to the regional population. This assumes these counties are similar, which is a reasonable assumption.

Projections of mortality rates are uncertain. Official estimates by the Social Security Administration have tended to underestimate improvements in mortality due to medical advances and public health (Canudas-Romo et al., 2016). We used rates that may have improved the accuracy of estimates by incorporating expert clinical and public health judgment. The available age bands and disease categories for future death rates were broader than those used in the ITHIM model. This may have the effect of underestimating the age-sex-and cause specific deaths and DALYs in 2040. Also, we applied the annual percentage change in death rates to not only deaths, but to DALYs, whose annual percent change may not follow death rates.

In contrast to previous versions of ITHIM, we made an additional assumption that, for the ambitious scenarios, active transport would only substitute for car miles driven on local roads and arterials. This is a reasonable assumption given that pedestrian and bicyclist travel on highways is rare. Because cars striking pedestrians, bicyclists and other motorized vehicles, including other cars, comprise the largest share of traffic collisions, reducing car VMT on these local and arterial roads leads to large reductions in overall injuries. In the ambitious scenarios, we substituted car miles across arterials and local roads in proportion to the baseline ratio of local to arterial car miles rather than constraining car miles to the baseline levels for local roads and arterials. This approach reduced but did not eliminate car access on local roads in the Cycle and Blend scenarios.

Age and gender strongly influence travel patterns and health outcomes; however, unlike in some previous analyses, we did not assume that as active travel becomes more prevalent there would be greater gender equity and a greater share of active travellers in older age groups (e.g. less prominent peak of bicycling among young males). In sensitivity analyses, this flattening increased the population attributable fraction by 1% to 2% as older individuals engage in active transport. Projections of disease rates suggest that older people may be healthier in the future, so there may be greater potential to be active.

Compensatory balancing of travel-related and non-travel related physical activity (activity substitution) is not included. Given the large percentage of the population that reports no daily physical activity, activity substitution may have its largest impact in the small percentage of the population that is highly active, who, with non-linear dose response curves have the least to gain. Recent evidence suggests that the introduction of transit may promote physical activity in the least active without inducing activity substitution in the previously active (Panter et al., 2016). The scenarios assumed that meeting the U.S. Surgeon General recommendations was achieved exclusively from an increase in active transport, but we recognize that leisure-time physical activity contributes to this goal.

Modeling the health impacts of air pollution using chemical transport models (Fresno Council of Governments, 2014, Institute for Health Metrics and Evaluation, 2014) is technically challenging, resource intensive, and was beyond the scope of the present research. Our projections of the annual physical activity benefit of the SCSs (1,670 in 2040) are almost twice those projected for fine particulate air pollution (880 in 2030) based on GHGE reduction strategies in all California sectors (industry, electric utility and natural gas, agriculture, on-road vehicles, and other mobile sources) (Zapata et al., 2012).

A thorough exploration of the policy implications of our findings was beyond the scope of this work. However, readers are referred to other research that addresses the roles of MPOs and local government in implementing SB375 (Niemeier et al., 2015) and the transportation component of health in all policies (Maizlish et al., 2013, Sacramento Area Council of Governments, 2016, Wernham and Teustch, 2015).

## 5 Conclusions

Active transport strategies that promote ambitious levels of walking, bicycling, and transit will significantly improve population health in California. Transportation planning agencies are incorporating increasing levels of active transport, but their preferred scenarios fall short of optimum levels that, in theory, do not increase the travel time burden. Strategies that emphasize bicycling and transit will have additional benefits in curbing the carbon emissions from cars as the California population grows. In ambitious active travel scenarios, there is an overall decrease in burden of chronic disease and injuries, whose benefit is dependent on the safety-in-numbers effect. Achieving large increases in active travel and realizing the safety-in-numbers benefits is likely to require spatial separation of motorized traffic and active travelers (separated bike and/or pedestrian paths), reducing maximum speeds on local and arterial roads, and greater enforcement of dangerous driving (British Medical Association Science and Education Department and the (Board of Science), 2012; Teschke et al., 2012; Aldred et al., 2017; Buehler and Dill, 2015).

The profile of active transport as a strategy for carbon mitigation may be enhanced by including explicit carbon targets tied to levels of walking, bicycling, and transit in regional and state plans.
